# ETIOPATHOGENIC, EPIDEMIOLOGIC AND CLINICAL-THERAPEUTIC COMPARISON OF NON-HODGKIN’S LYMPHOMA AND KAPOSI’S SARCOMA

**DOI:** 10.1590/0102-672020200002e1521

**Published:** 2020-11-20

**Authors:** Andy PETROIANU, Luiz Ronaldo ALBERTI, Vanessa Lacerda ORSI, Flávia Costa Teixeira VIANA, Carolina Braga MOURA

**Affiliations:** 1 Surgery, Federal University of Minas Gerais, Belo Horizonte, MG, Brazil; 2 Surgery of the Digestive System, Felício Rocho Hospital, Belo Horizonte, MG, Brazil; 3 Medicine, Faculty of Medical Sciences of Minas Gerais, Belo Horizonte, MG, Brazil; 4 Medicine, Faculty of Health and Human Ecology, Vespasiano, MG, Brazil

**Keywords:** Lymphoma, non-Hodgkin, Sarcoma, Kaposi, Etiology, Population characteristics, Linfoma não Hodgkin, Sarcoma de Kaposi/etiologia, Características da população

## Abstract

**Background:**

Non-Hodgkin’s lymphomas (NHL) are primary neoplasms derived from lymphocytes, and Kaposi’s sarcoma (SK) is a multicentric disease of viral etiology and is associated with HIV.

**Aim:**

To study the etiopathogenesis and clinical characteristics of NHL and KS, describing their mutual factors.

**Methods:**

This retrospective investigation was performed on 101 medical charts. The patients were studied according to their age, gender, and HIV-positivity, following the PRISMA guidelines. The characteristics of the tumors and comorbidities were analyzed according to their age and lymphatic metastasis.

**Results:**

The mean age of the patients ranged between 15-87 years for NHL and between 25-54 for KS, but the age of patients with NHL associated with HIV did not surpass 34 years. The ratio male: female was 1,8:1 for NHL, but only men presented KS. HIV-positivity was found in five patients with NHL and in 14 with KS. The stages of NHL were: I (21%), II (18,4%), III (26,3%), and IV (34,2%), but KS were found only at III (40%) and IV (60%) stages. The lymphatic metastases were positive in 62 patients NHL and in four with KS. HIV-positivity occurred in 60% of patients with NHL and in 50% with KS.

**Conclusion:**

The HIV seropositivity was revealed for most of patients during the NHL and SK propaedeutic and none of them present clinical manifestations of AIDS. NHL associated with HIV was found only in young patients. NHL and KS patients have similar epidemiological, clinical, and therapeutic characteristics.

## RESUMO

**Racional:**

Os linfomas não Hodgkin (LNH) são neoplasias primárias derivadas de linfócitos e o sarcoma de Kaposi (SK) é doença multicêntrica de etiologia viral, ambas associadas ao HIV*.*

**Objetivo:**

Avaliar características clínicas dos LNH e SK, relacionando fatores etiopatogênicos mútuos.

**Métodos:**

Foram avaliados retrospectivamente 101 prontuários. Os doentes foram analisados quanto a idade, sexo e soropositividade para o HIV, de acordo com o PRISMA guidelines. Os tumores foram classificados por estadiamento, presença de linfonodos regionais invadidos e tipo celular.

**Resultados:**

A idade variou entre 15 e 87 anos para o LNH e 25 a 54 anos para o SK, mas a idade dos pacientes com LNH associado com o HIV não ultrapassou 34 anos. A proporção homem: mulher foi de 1,8:1 para o LNH, enquanto SK foi registrado apenas em homens. A soropositividade para o HIV ocorreu em cinco pacientes com LNH e 14 com SK. A invasão de linfonodos regionais foi positiva em 62 com LNH e quatro com SK. Os linfomas foram 27,9% de baixo grau, 17,4% de grau intermediário e 12,8% de alto grau. A soropositividade para HIV, foi diagnosticada durante a propedêutica do tumor em 60% dos pacientes com LNH e 50% dos com SK.

**Conclusão:**

A maioria dos pacientes portadores de HIV descobriram a soropositividade durante propedêutica para LNH e SK, sem manifestações clínicas de AIDS. Todos os pacientes com LNH associado com o HIV eram jovens. Pacientes com LNH e com SK apresentam características epidemiológicas, clínicas e terapêuticas semelhantes entre si.

## INTRODUCTION

Non-Hodgkin’s lymphomas (NHL) are primary neoplasms derived from lymphocytes, which are manifested as solid tumors in lymph nodes, oropharyngeal structures, spleen, gastrointestinal submucosa, liver, bone marrow and lung^1^ ^,^ ^19^. Regardless of the tumor nature, all forms of lymphoma have the potential to spread to tissues of the mononuclear phagocytic system. In a more advanced stage, blood involvement creates a picture similar to that of leukemia^19^. Surveillance, Epidemiology, and End Results data from the National Cancer Institute, showed that until 1980 the incidence of NHL increased annually by 3-4%; however, in the following decades, this growth was reduced to less than 1%^2^ ^,^ ^3^. According to the Northern California Cancer Center, in the United States, its incidence remained stable in children but continued to increase among Caucasians aged 15-24 years (2-3% per year), women aged 25-54 years (1-6 % per year) and African-Americans over 55 years old (2-4% per year)^5^. Despite the controversies in relation to the classification of NHL, the most accepted criteria are the Working Formulation, which has low, intermediate, and high-grade lymphomas, with a ten-year survival prognosis of 45%, 26%, and 23%, respectively.

Kaposi’s sarcoma (SK) is a multicentric disease of viral etiology^4^ ^,^ ^5^ ^,^ ^6^, originating from endothelial cells and pericytes, with four known forms, classic or Mediterranean, endemic or African, post-transplant and epidemic or associated with HIV. The epidemic type is the most common and presents clinically with red/purple lesions, which can reach mucous surfaces, lymph nodes, salivary glands, and viscera. Although clinical responses differ between the epidemiological forms of KS, new findings indicate a common infectious agent as the cause of the tumor in immunocompromised patients. Recent research points to the KSHV/HHV8 virus as being the main responsible for this disorder^4^ ^,^ ^5^ ^,^ ^6^. The simultaneous occurrence of KS and lymphoproliferative diseases in the same patient rises the possibility of mutual etiopathogenic factors^3^. The first published case of an association between these diseases dates back to 1920, when Cole and Crump described the coexistence of KS and chronic lymphocytic leukemia. In 1993, Lone and Greenwood published a case of KS complicated by fungal mycosis. However, a study carried out at the Institute of Dermatology in Milan found only six associations in 250 patients^7^ ^-^ ^10^.

Patients with HIV are at high risk of developing NHL and SK^4^. In Europe, the prevalence of NHL associated with AIDS increased from 3.6% to 5.4% between 1994 and 2000. The antiretroviral therapy reduced the incidence of NHL. Burkitt’s tumor and immunoblastic lymphomas have been classified as AIDS-related diseases since the early 1980s^6^ ^,^ ^11^ ^-^ ^13^.

The association between NHL and HIV is less common in Africa than in most developed countries. In the United States, the prevalence of NHL in the African-American population is lower than in Caucasians. The small number of NHL patients in Africa may be ascribed to the early high mortality from infectious diseases and malnutrition, in general before the age of 40, when this tumor starts to be present^13^ ^,^ ^14^ ^,^ ^15^ ^,^ ^16^. 

Patients undergoing kidney transplantation, whose immune system is depressed by immunosuppressants, develop NHL 40 to 100 times more than the general population^6^. Several viruses, such as HIV, have also been linked to these lymphomas^7^ ^-^ ^9^ ^,^ ^11^ and to adult T-lymphocyte leukemia.

There are environmental risk factors found in pesticides and herbicides, mainly 2,4-D, as well as in toxic industrial substances. Workers on those products present a higher incidence of NHL than in the general population^17^ ^,^ ^18^. However, the role of toxic substances in the etiopathogenesis of NHL has not been established.

The aim of this study was to verify the epidemiological, clinical, and therapeutic characteristics of patients with NHL and KS in a university hospital of clinics.

## METHODS

This study was carried out in accordance with the recommendations of the Declaration of Helsinki and Resolution 196/96 of the Ministry of Health (Brazil) involving human research, and it was approved by the Ethics Committee of the Federal University of Minas Gerais under protocol 256 / 2014.

This is a retrospective study of 101 patients treated between 1997 and 2005 for NHL and SK at the Hospital das Clínicas of the Federal University of Minas Gerais, Belo Horizonte, MG, Brazil. Their diagnosis was confirmed by anatomopathological exams. All these patients were followed-up until 2017, and the presence of HIV was investigated to verify a relationship between these tumors and HIV infection.

Patients were identified according to age and gender. The tumor staging took into account its type, location, and presence of metastatic lymph nodes. Previous cancer treatment, other metastases, and the post-treatment follow-up were also investigated. 

The results were compared, according to the PRISMA guidelines, using the chi-square test. Differences corresponding to p<0.05 were considered significant.

## RESULTS

The 101 consecutive charts were related to NHL (n=86) and SK (n=15), 70 were men and 31 women, with a male prevalence of 2.3:1. The patients’ age ranged between 15 and 87 years, with a median of 50.2 years (Table 1).

**TABLE 1:** Age distribution of patients with non-Hodgkin’s lymphoma and Kaposi’s sarcoma

Age (years)	Non-Hodgkin’s lymphoma		Kaposi’s sarcoma	
	n	%	n	%
15 - 24	8	9.3	0	0
25 - 34	8	9.3	10	66.6
35 - 44	7	8.1	4	26.6
45 - 54	16	18.6	1	6.6
55 - 64	23	26.7	0	0
65 - 74	16	18.6	0	0
> 74	8	9.3	0	0
Total	86	85.14	15	14.86

n=number of patients

Seropositivity for HIV was present in 20 patients, including all the patients with KS and five (5.8%) with NHL. The AIDS diagnosis was made during the tumor propedeutics in 12 (60%) patients. The HIV seropositivity was found mainly in patients between 25 and 34 years old.

Diagnosis of lymph nodes metastases was made in 66 cases and, in eight patients, the results were inconclusive. The stages of the neoplasms were stage I in 17 cases, stage II in 13 cases, stage III in 24 cases, stage IV in 33 cases, and undefined in 14 cases. Lymphomas were low grade in 24 (27.9%), intermediate grade in 15 (17.4%), and high grade in 11 (12.8%) patients. In the 51 (50.5%) cases, it was not possible to establish the stage as intermediate or high (Tables 2 and 3).

**TABLE 2:** Location of non-Hodgkin’s lymphomas

Location	n	%
Inguinal lymph nodes	15	17.6
Cervical lymph nodes	12	14.1
Axillary lymph nodes	7	8.2
Bone marrow	7	8.2
Stomach	7	8.2
Skin	7	8.2
Intestine	6	7
Face	6	7
Mediastinal lymph nodes	6	7
Spleen	3	3.5
Liver	2	2.3
Retroperitoneal lymph nodes	2	2.3
Unspecified lymph nodes	15	17.6

n=number of patients

**TABLE 3:** Kaposi’s sarcomas location

Location	n	%
Lower members	7	46.6
Head	5	33.3
Upper limbs	5	33.3
Chest	4	26.6
Abdomen	3	20
Oropharynx	3	20
Disseminated	3	20
Inguinal region	2	13.3
Axillary region	1	6.6
No location	2	13.3

n=number of patients

In 71 patients metastasis was not registered, while in the other 30 patients, the metastases were present in liver (9%), lung (9%) and brain (6.8%, Tables 2 and 3)

Curative intention treatment was made in 57 patients and palliative one, in 44 patients. Chemotherapy with cyclophosphamide, vincristine, adriamycin, and prednisone was indicated for most patients. This treatment was efficacious in 35 (61.4%) patients. Recurrences occurred in 20 cases, between two months and eight years (mean of 22.8 months) after the end of treatment.

## DISCUSSION

The high prevalence of men with NHL described in the literature was also found in this work when compared with KS^6^ ^,^ ^19^ ^-^ ^21^. Although NHL is more common in the elderly, in this study, when this disease was associated with the presence of HIV, the age has not surpassed 34 years. There are no data to indicate the role of HIV in the etiopathogenesis of NHL, considering that this virus was not found within tumor cells. However, immunity disorder and cytokine dysfunction certainly contributed to the occurrence of this disease in early age^15^.

In the etiology of KS, the presence of the KSHV / HHV8 in all patients with indicates the importance of the role played by the sexually transmitted virus. This association is reinforced by the manifestation of KSHV / HHV8 in only 30% of HIV positive patients and in less than 3% of HIV positive hemophiliacs. In children, KS associated with HIV is due to the transmission of KSHV / HHV8 through the mother^18^. According to European data, 4% of the population with AIDS have NHL with HIV seropositivity, even without clinical manifestation^10^. In this study, 57.8% of patients HIV seropositive did not have any clinical manifestation of AIDS and did not even know they were HIV seropositive.

According to the literature, 80% of NHL patients present lymphadenopathy, mainly in the cervical location^21^. In this series, 76% of the patients had lymphadenopathy, but the prevalence was in the inguinal site (Table 2). According to the National Cancer Institute, 31% of patients with poorly differentiated lymphocytic NHL type have liver disease or metastasis, confirmed by percutaneous biopsy^20^. However, in this study, only 2.3% of patients presented liver tumor at ultrasound and tomography search (Table 2).

Previously, mediastinal adenopathy was described in 18% of NHL cases, pleural effusion in 8%, lung metastasis in 3%, and brain metastasis in 10% of cases^20^. Otherwise, in this study, no thoracic or brain metastasis or tumor was found.

Although most of NHL were of low grade, their stage was advanced (III and IV). This data reveals the systemic character of this disorder since the beginning of its clinical manifestations. Radiotherapy is the treatment of choice for low-grade NHL, mainly on head and neck location, with a survival rate greater than 10 years in up to 60% of patients^21^. Chemotherapy using CHOP is the best treatment for the widespread disease, leading good long-term results^22^.

## CONCLUSION

The HIV seropositivity was revealed for most of patients during the NHL and SK propaedeutic and none of them present clinical manifestations of AIDS. Although NHL associated with HIV was found only in young patients, it was not possible to identify the role of HIV in the etiology of NHL. NHL and KS patients have similar epidemiological, clinical, and therapeutic characteristics.
